# Beliefs about self-care by pregnant women belonging to a population group in Monteria, Córdoba, Colombia.

**DOI:** 10.1192/j.eurpsy.2023.1904

**Published:** 2023-07-19

**Authors:** E. P. Ruiz Gonzalez, J. D. Velez Carvajal, D. A. Guzmán Bejarano, A. L. Malluk Marenco, J. Agudelo Jiménez

**Affiliations:** 1 Universidad Pontifica Bolivariana, Montería; 2 Universidad Libre, Bogota, Colombia

## Abstract

**Introduction:**

Scientific disciplines recognize that pregnancy not only refers to the biological dimension. It also constitutes a social category, since sociocultural matrices have implications on what is conceived as the state of gestation (Noguera & Rodríguez, 2008). In this sense, cultures develop protocols to guide the actions of pregnant women and their loved ones regarding self-care during pregnancy in order to contribute to the well-being of mother and child (Carmona, Hurtado and Marín 2007). In this context, the belief category becomes relevant as a form of understanding the ways in which we appropriate reality and intervene it (Peirce, 1903).

**Objectives:**

To analyze the beliefs that a group of pregnant women belonging to a population group from Montería (Córdoba, Colombia) have about taking care of themselves.

**Methods:**

Approach: qualitative. The sample was defined by saturation, for a total of 15 pregnant women affiliated to the Mocarí Hospital in the city of Montería, Córdoba. Instrument: semi-structured open interview; content analysis technique through AtlasTi. Emerging categories: a) care during pregnancy; b) relationships with others.

**Results:**

Main belief: Pregnant women need to take care of themselves physically and psychologically, for which it is necessary to have parents, siblings and partner’s support. Care is based on healthy nutrition, physical activity and mental health prevention. It is assumed that self-care is important for the well-being of mother and child. The importance of the family support networks’ participation is also recognized.

**Image:**

**Figure:**
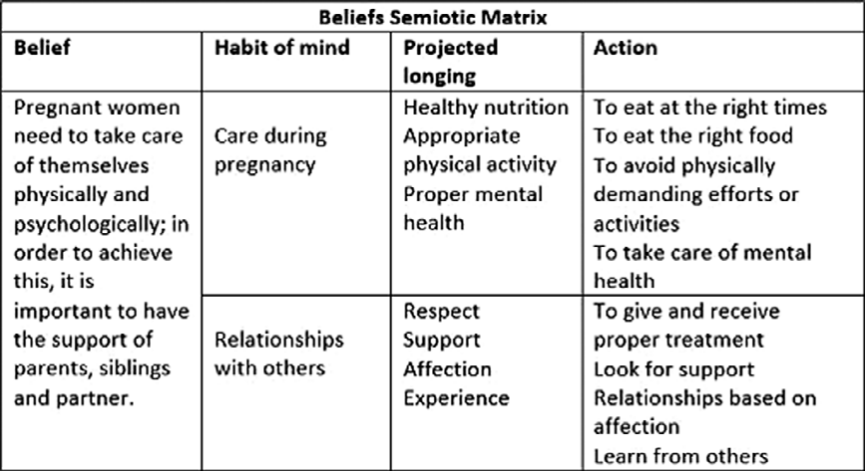

**Conclusions:**

According to the approaches stated/developed by Peirce (1903), beliefs have implications on the way we behave and intervene in reality. Mental habits function as a link between belief and concrete action. For this research, the beliefs that arise from the sociocultural matrices of the pregnant women are evidenced in their concrete actions.

**Disclosure of Interest:**

None Declared

